# Rethinking local welfare: a network analysis of an Italian case study

**DOI:** 10.3389/fsoc.2025.1690488

**Published:** 2025-12-19

**Authors:** Silvia Carbone

**Affiliations:** Department of Political and Legal Sciences, University of Messina, Messina, Italy

**Keywords:** infrastructural network, local welfare, networks analysis, social network analysis, social policies

## Abstract

**Introduction:**

The hypothesis underlying this research is that a participatory/co-design research experience, within a specific territorial context, can foster interaction between the social actors involved, enabling interesting mechanisms for transforming the infrastructure network.

**Methods:**

We applied social network analysis methodology. We evaluated two interaction systems and observed how actors connect and how behavior is influenced, with an impact on the infrastructure network.

**Results:**

Our results show that a participatory research/co-design experience can create an engagement strategy and a network infrastructure capable of implementing local wellbeing.

**Discussion:**

We can conclude by stating that the Mapping project was able to actively involve, in both cases, organizations and associations/cooperatives, consolidating and sharing a common vision on community planning. A local welfare system does not exist in isolation or as something simply imposed from above.

## Introduction

The territorial dimension is now an essential key to understanding the growing differentiation of welfare systems, fuelled by decentralization processes and the emergence of new sub-national decision-making spaces. In this perspective, as Ferrera (2007) also points out, the governance of social policies is increasingly taking the form of a multi-level system, in which the multiplication of public and private actors involved is not limited to the mere implementation of interventions, but contributes directly to the institutional design of the measures themselves. These developments have led to the formation of new territorial configurations of welfare, characterized by differentiated models and a growing intertwining of national, regional and local competences in order to provide social responses on a territorial basis (Moreno and McEwen, 2005; Ferrera, 2007). In this sense, the call for local welfare therefore does not just end with the invocation of a takeover of market efficiency and regulatory forms. But it means directing it toward a principle of subsidiarity that points at the reconstruction of that base of practical resources and relational skills, that is, that aims at the social infrastructure on which an inclusive and democratic local welfare is based. In recent years, as a result of the importance of subsidiarity and the local dimension of social policies (Kazepov and Cefalo, 2020), local social innovation practices have become increasingly strategic, having to use the pro-social resources of the territories to deal with the increase in needs and the reduction in the responsiveness of institutional welfare (Ferrera, 2007). Through these local practices, a new capital of services and networks is built, capable of responding, in an innovative way, to protection needs, also promoting the strengthening of the social issue, and generating new development paths. It is within this framework that the theme of social infrastructuring, which has become increasingly central also in reflections on the future of local welfare systems, takes on importance. In this perspective, social infrastructuring can be defined as the development of intangible structures, i.e., relational networks, that provide the opportunity to connect a multiplicity of places and subjects, in order to make them know each other, dialogue and work together for the common good [Fondazione con il Sud (FCS), 2021]. Thus, social infrastructures do not only consist of space and artifacts dedicated to citizenship, but above all of networks of interconnection. These constitute opportunities for the exchange of social capital, skills, ideas and practices for the construction of further networks (Larkin, 2013). Networks thus represent the socio-relational and organizational foundations that support the functioning of local welfare (Star, 1999), and the embedding of social innovation (Latham and Layton, 2022). Applied to the development of local welfare, this perspective emphasizes the link between social issues, human capital and endogenous development, in order to build an infrastructure of networks in the territory that facilitates collaboration between actors with pro-social skills, and who take responsibility for acting to generate development, in a participatory logic. This undertaking does not end with the construction of the network, but must enhance the social aggregation process of activating an endogenous development vision, and its implementation as a participatory practice of transforming the territory. This process must then be able to destabilize the pre-existing situation, and thus generate political growth in the territory (Luongo et al., 2022). If, on the one hand, it is important to reactivate the protagonism of the territories in being themselves the promoters of their own development, on the other hand, it is necessary to reconsider all the interventions, not only as solutions to buffer uncomfortable situations, but also as practices oriented toward the overall growth of the territory. Therefore, the conceptualization and practices of local welfare infrastructure overlap as horizons of action. In fact, both are based on a participatory logic that activates an incremental path of experimentation, learning and local transformation, oriented toward social cohesion. The hypothesis underlying this research is that a participatory research/co-design experience, as “Resource Mapping” experience (Kumar, 2002), within a specific territorial context, has favored interaction between the social actors involved, enabling interesting infrastructural network transformation mechanisms. The project was named Mapping (in abbreviated form) because, basically, it refers to the approach used, originally based on Kumar's classic guide to community participation (2002) and incorporating Gangarova's experience (2020) on community mapping (asset mapping), consisted of identifying and representing the community's assets: people, associations, spaces, skills, public places, and social networks. This method emphasized existing resources rather than just needs. Resource mapping, as a participatory exercise through which participants identified, located, and visually represented the resources available in the portion of territory under consideration, made it possible to highlight and spatially distribute natural, material, infrastructural, and social resources with the aid of actual paper and digital maps.

However, the need to build a heterogeneous, permeable and continuously expanding territorial network, capable of activating the instances of transformative protagonism for local welfare on the part of its components, entails the need to clarify what are the constituent foundations and perimeters of this territorial dimension. Put another way, what is the concrete operational dimension on which to base local welfare action? In this contribution, the territorial dimension will be privileged, but an attempt will also be made to illustrate how it interacts with the multiplication of actors. Considering the territory from a geographical point of view, looking at the institutions and proximity relations of a given territory (Zamengo and Valenzano, 2018), although it helps us to move away from the ideological connotation of territory/local community, nevertheless presents conceptual risks, since each territory is endowed with its own specific reality. Therefore, it would be naive to think that from mere spatial sharing, the experience and attitude of working together also follows, just because certain organizations or people operate in the same spatial context. In many territory, there is conflict caused by the difficulty of establishing genuine collaboration between actors, which is more than simply joining a network, and this often leads to mistrust, disinterest in practices and self-referentiality (Rizzuto, 2020). This closed-mindedness primarily affects the positive potential of interventions, both in terms of beneficiaries and for the organization itself. Furthermore, another difficulty may arise from not considering the multiple interpretations that different professional points of view can give to situations, needs, objectives and, consequently, intervention strategies (Triani, 2014).

Instead, we consider it need to recognize that local welfare is the outcome of a construction process that is expressed both in the relationships on the territory and in the conflicts between the actors involved (Bartlett and Vavrus, 2017). It would be important to value the richness of different points of view and establish a critical dialogue aimed at finding the synthesis that best represents the situation, which will become more understandable in its multidimensionality. To this end, it is better to adopt an attitude that embraces diversity, including with regard to the specific constraints to which each actor is subject in terms of established practices, resources, bureaucratic constraints, etc.

From this perspective, it's not possible to consider the territory as an internally homogeneous identity. Instead, it is inevitable that it is plowed by different competitions and contrasts between different social groups. It should therefore be avoided to automatically apply predefined categories and goals to the territory, and its components, because these can indeed be constituted in a different way and in other conditions. A local welfare can thus find its raison d'être in a territorial development project co-constructed with those who decide to “be part of it,” i.e., with those who voluntarily assume responsibility for implementing a participatory and shared transformative project that defines the horizon of meaning of the territory's actions (Rossi-Doria, 2020). This assumption of responsibility is therefore not based on the territorial imposition toward a given affiliation, but is based on the recognition of a road to be taken together, voluntarily and intentionally (Meo, 2022). In summary, the framework within which to give meaning to the action of the territory should therefore abandon the romantic references that reduce it to an idealized entity, and seek the strengthening of social ties, without neglecting the safeguarding of the freedom of individual actors, in order to build a territorial vision in which social base, perimeters, aims and actions are defined in a flexible way, through a continuous process of social mediation. After having outlined the characteristics of the territory, and having identified its transformational perspective, it remains to reflect on how these intentions can be operationally translated in the field. The first critical issue that emerges is to establish an attitude of real collaboration between the actors involved. This is to be understood as something more than simply joining a network, or simply carrying out its own activities of competence within a larger system (Rizzuto, 2020). The second risk is the failure to take into account the multiplicity of readings that different professional points of view can give to situations, needs and objectives, and therefore to intervention strategies (Triani, 2014). It is need to be able to implement a critical dialogue between the actors involved, useful for the search of synthesis between the different parties, representing the situation in a multidimensional way from multiple perspectives. Through the communion of intentions and practices between the various actors, and a dialectical and prospective confrontation, misunderstandings can be resolved and the actors can be stimulated to overcome the tendency to defend their respective readings. In short, the project must become an arena of participation, modeled on a governance architecture suitable to promote communication and exchange between the different actors, with a view to exploiting the operational potential, mutual learning and strategic alignment (Rotondi, 2019). Finally, the sharing of the decision-making process is a characteristic feature of horizontal networks, because it can facilitate the activation of a widespread role and essential for the development of local welfare. The latter must constantly move between formal and informal, connecting institutions, organizations, and building a relational infrastructure to promote the participation of all (Valenzano, 2019). Local welfare, discussed in this article, which examines an Italian case study, shows precisely this new horizon for Italian local welfare, which seeks to move beyond the complexities of a local welfare system undergoing transformation. In recent years, despite financial constraints, changes in social demand and the redefinition of the role of municipalities, a new impetus has been found in the reform of the Third Sector (Legislative Decree 117/2017) and in the spread of local co-design practices, ensuring elements of social innovation and collaborative governance aimed at building a true community welfare system. When analyzing local welfare in Italy, it is worth noting that, over the last 20 years, municipalities have gradually become the central actors in the planning and management of social policies. Accorinti (2008) had already highlighted how the transformation of local welfare has been characterized by the emergence of a plurality of actors—in particular the Third sector—called upon to collaborate with local authorities in the provision of services, giving rise to mixed welfare systems based on forms of public-private-social partnership. In this perspective, the Third sector no longer plays a merely integrative role with respect to public intervention, but becomes a co-protagonist of local governance, contributing to the design of services and the definition of needs through increasingly close interaction with municipalities. Giovannetti et al. (2014) confirm and explore this evolution, showing how local welfare is undergoing a phase of structural redefinition linked to the growing complexity of social demand—influenced by an aging population, increasing inequalities and the economic crisis—generating a contrast between reduced public resources and the weakening of the operational capacity of local authorities. This situation, defined by the authors as an identity crisis of municipal welfare, in which municipalities continue to be the point of reference for citizens, despite often operating with insufficient organizational and financial capacity, is overcome through the adoption of tools more consistent with contemporary complexity, opening up to generative and territorial welfare models that enhance social resources, local networks and community capital. The impact of the pandemic has further accelerated this transformation of local welfare systems in Italy, clearly highlighting the limitations of vertical and sectoral models and increasing attention to these integrated approaches, oriented toward territorial proximity and collaboration between social, health, educational and community services. The National Recovery and Resilience Plan (PNRR) also reinforces this direction, investing in the areas of home care, disability, social inclusion and housing, while at the same time providing for the activation of local networks and stable partnerships as conditions for access to funds. All this increases the importance of cooperation between municipalities, Third sector organizations, health institutions and community actors, pushing toward a welfare system oriented toward proximity, integrated care and social co-responsibility. This approach, which values the skills of the Third sector in defining needs and designing social policies, appears to be in line with the international collaborative models described by Osborne (2010) and Emerson et al. (2012), which highlight how collaborative governance increases the effectiveness of public services and the resilience of communities. This need to move beyond a model based on the mere provision of services in favor of an approach that strengthens governance functions and integration between services and territorial coordination, involving both public and private social actors in a structured manner, as Kettl (2015), shows that collaboration between public bodies and private and non-profit organizations is fundamental to addressing the growing social complexity in urban contexts, and that public-private-social partnerships are essential to ensuring social cohesion and the sustainability of local services, even in Nordic and Mediterranean contexts (Albæk et al., 2019).

## Methodology

The objective of this research is to assess how the two systems (Case1, for District III the Camaro-Bisconte quarter; Case 2, for District IV the Gravitelli-Montepiselli quarter) of interaction analyzed connect actors and influence their behavior, affecting the networked infrastructure. This case study approach will provide, as Stake (2008) states, privileged information with respect to the complexity of local and specifically situated welfare (situational uniqueness). We therefore adhered to the qualifying characteristics of the case study as described by Stake (1995): (a) particularity, i.e., we focused on a specific case, in order to understand its unique dynamics and peculiarities; (b) research background, we studied the case within its natural context, considering all the social, cultural, economic and historical dynamics that influence the behavior and experiences of the case studied; (c) process, which is achieved through the development of the interaction between researcher and field of observation; (d) meaning, we explored and understood the meaning attributed by the people involved. These features are crucial to ensure that the case study provides a deep and comprehensive understanding of the case being examined, rather than just a superficial description.

### Research background

We decided to start from some data that emerged as a result of a first-year training course of the Bachelor of Science in Social Work at the University of Messina (cohort 2023/2024), which envisaged a participatory and situated teaching/research experience, based on the use of a participatory methodology, giving rise to a field research we called Mapping (for a more in-depth study see Carbone, 2025). As mentioned above, the Mapping project is based on two approaches and focuses on two strategic areas. The approaches used are originally based on Kumar's classic guide to community participation (2002), which incorporates Gangarova and von Unger (2020) experience of community mapping (resource mapping), enabling the identification and representation of community resources (people, associations, spaces, skills, public places and social networks). The strategic areas are pedagogy and local development. In fact, the mapping project was first used as a participatory tool to try to reduce the gap between theory and practice for students (Everitt et al., 1992), and secondly to increase social co-responsibility among all local actors, establishing project alliances to respond positively to needs, mobilizing resources within the community itself and strengthening the link with informal networks in the area (Zhu and Baylen, 2005). In involving students (63), teachers/researchers, educational institutions, social workers, political representatives, and community, we realized and shared a process of democratic co-construction of knowledge starting from the shared territory (District III and District IV of the Municipality of Messina) and based on the valorization of the different perspectives of each of the participants. This activity started in October 2023, and ended in April 2024. We divided the activity into 10 afternoon classroom meetings (one per week on average every 15 days), 3 h each, and 70 h of field research and group study activities. Classroom activities had compulsory attendance, and the coordination and monitoring of the activity was conducted by the researcher. The aim was to build a shared training space, to create the conditions for situated learning. Students have become the focal point of that network that unites actors of different natures in the field of social services. A network where each node links the others, in a sharing of knowledge and experience capable of enhancing the experiences and specificities of a territory. The students, guided by the researcher, carried out participant observations in the area, visiting the locations and observing the existing conditions and social interactions that take place in those spaces, recording data, reflections and considerations in their logbooks; conducted focus groups with teachers within partner schools, attended by head teachers, school managers and teachers responsible for different areas (disability, bullying, dropouts, etc.); identified and created a list of stakeholders in the two districts and constructed a grid of questions for in-depth interviews that encouraged discussion of the issues, needs and services present in the area; created a questionnaire/form (to be administered near the school) and a questionnaire for the general population (to be administered in the neighborhood/districts) and stakeholders to gather information on the interactions and social networks in which they are involved and from which they draw support and help (this tool facilitated the emergence of information concerning local social networks); creation and sharing with all participants of maps showing the locations of reference points consisting of resources, places, services and actors that emerged from the interviews and other data collected. In this initial data collection phase, the researcher, in training, guiding and constantly monitoring the students' work, highlighted the intertwining of public services and the Third Sector, in order to describe the informal support that complements the formal support, analyzing the relationships, exchanges and contacts within the networks.

The start-up of the Mapping project was also made possible thanks to the participation of the Headmasters (and respective teachers in charge) of the Comprehensive Institutes of the two Circumscriptions examined, with whom a formal agreement had been signed prior to the start of the project. The following were involved in the project: two Comprehensive Institutes (I.C. La Pira Gentiluomo di Camaro in the 3rd Circoscrizione; and I.C. Paino/Gravitelli di Gravitelli in the 4th Circoscrizione), the Juvenile Court (USSM), the Local Health Authority (ASP), other Associations and Third Sector Cooperatives, active in the area, which offer various local welfare services, as can be seen in Table 1.

**Table 1:** Role and services provided in local welfare by the actors participating in the research.

**Type of service**	**Name**	**Interventions/services**	**Collaboration/governance modes**
Social assistance and community services	Lions club	Community services, food drives, free health screenings, solidarity projects and charity initiatives	Co-design between schools, municipalities, and NGOs; mentoring and tutoring by associations
Health and social-health services	ASP	Psychological counseling services, home care for patients, community health prevention, and a child neuropsychiatrist	Collaboration between local health authorities, municipalities, and non-profit organizations; citizen participation in treatment pathways
Services for disabled	Messina social city	Day centers, inclusive workshops, independent learning paths, dedicated transportation, after school	Co-planning between municipalities and social cooperatives/associations; shared management of services
Social inclusion and integration	Minor communities	Reception of migrants/refugees and young people in difficulty, residential centers, job/training placement programs, educational support for children at social risk	Local networks between municipalities, NGOs, and volunteer associations; co-programming of inclusion projects
Social health and social services, community services	Caritas/Chiesa San Paolino	Community services, food drives, free health screenings, solidarity projects and charity initiatives, reception centers	Co-design between schools, municipalities, and NGOs; mentoring and tutoring by associations, citizen participation in treatment pathways
Health and social-health services	Collereale	Hospitality house and kindergartens	Collaboration between local health authorities, municipalities
Social services for minors	USSM	Support and assistance to minors who commit crimes, individualized intervention plans, educational support for children at social risk	Collaboration between legal authorities
Social health and community services	GYM/ACR Camaro	Sports activities, volunteer and assistance services, social services for members and the community	Co-design between schools, municipalities, citizen participation in treatment pathways
Community services	Urban Park Camaro	Equipped green spaces, pedestrian paths, inclusive play areas, sports areas for workouts and calisthenics	Co-design between schools, municipalities, citizen participation in treatment pathways
Education and training	Save the children/bookstore store Bonazinga	Citizenship laboratories, educational support for children at social risk	Co-design between schools, municipalities, and NGOs; mentoring and tutoring by associations
Education and training	Association Ciclope	Educational support for children at social risk	Co-design between schools
Education and training	University of Messina/IC Piano/IC La Pira	Citizenship laboratories, educational support	Co-design between schools, municipalities, and NGOs; mentoring and tutoring by associations
Social services	Common	Day centers, recreation centers, inclusive workshops, independent learning paths, dedicated transportation, social housing, socioeconomic support, poverty relief packages, and rental assistance	Co-design between schools, municipalities, and NGOs; mentoring and tutoring by associations, citizen participation in treatment pathways

The two Comprehensive Institutes are located in fragile and difficult socio-territorial contexts, being popular areas of the cities and peripheral areas marked by educational poverty and the economic precariousness of families. For District III the Camaro-Bisconte quarter was chosen; for District IV the Gravitelli-Montepiselli quarter. Both quarters have witnessed a rapid increase in inhabitants over the years, following the spread of significant social housing, burdened by the presence of run-down structures. This has facilitated the settlement of low-income, jobless households. This can be considered one of the factors that has led to the process of cultural and social decay in these two parts of the city, together with the absence of adequate interventions to restore building decorum, the lack of well-maintained and available meeting and social places, and weak interventions to support families in difficulty. The working activities of the inhabitants of the two quarters are mainly linked more to the working-class, artisanal world, with occasional characteristics, and only rarely to the intellectual world, very few, in fact, are civil servants and professionals. The educational institutions, parishes and community centers in the two areas can represent an operational hub where old and new poverty can be encountered and confronted, offering the area innovative, informal and non-formal circumstances for growth.

**Figure 1:**
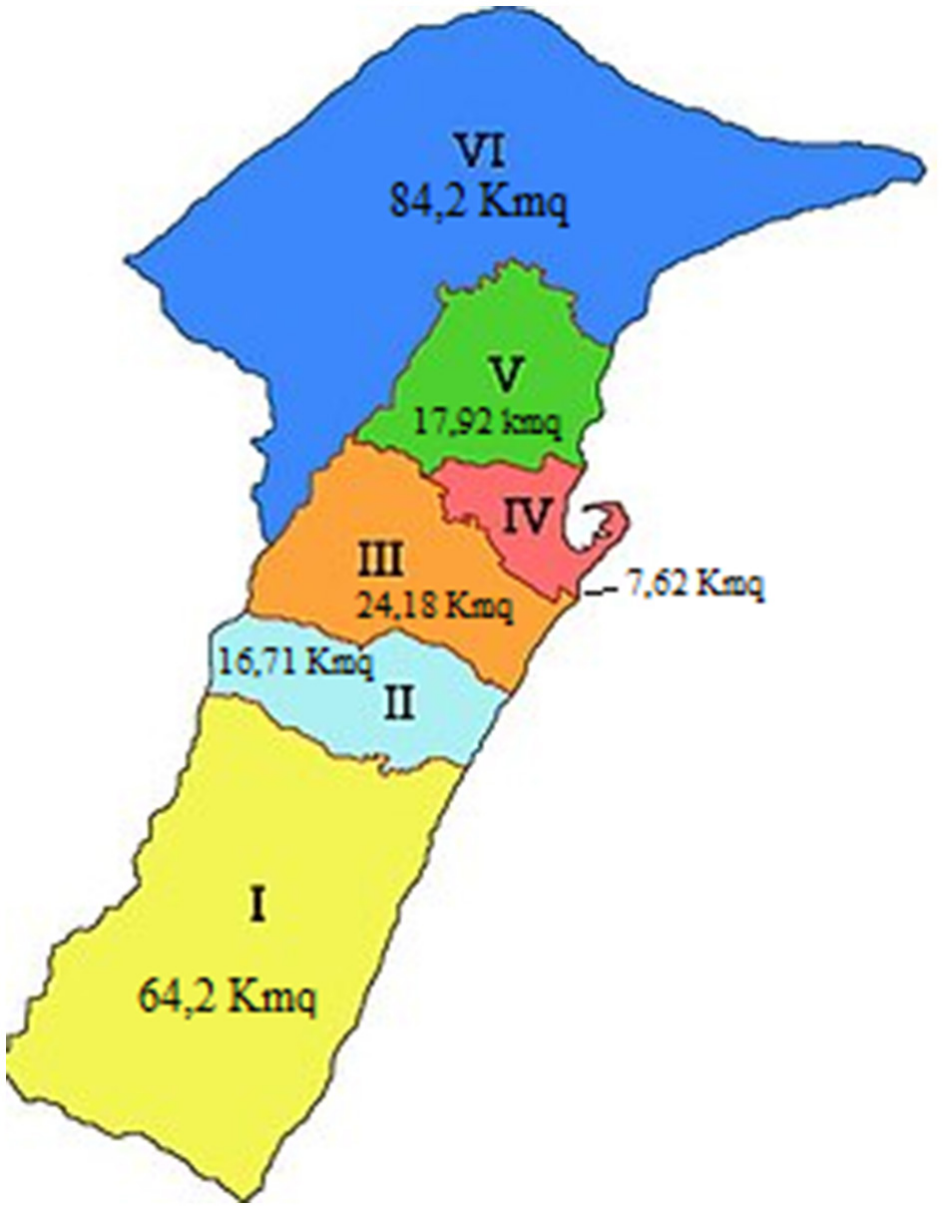
Messina districts and area in square kilometers.

Wanting to characterize in more detail, the areas of the two districts involved, from a spatial, social and economic point of view (Figure 1), it emerges that:

- Bisconte and Camaro are two neighborhoods and hilly areas west of the center of Messina. Camaro is historically linked to the mafia clan of the same name, while Bisconte is an area that includes the former Camaro powder magazine (built toward the end of the nineteenth century, it was used as a powder magazine, or storage facility for explosive materials, today, it is overgrown with weeds and littered with rubbish of all kinds) and the Masotto Barracks (disused for years now). Both neighborhoods are part of the same district and are characterized by a mix of houses and green areas. The entire area, which covers approximately 230,000 square meters and is crossed and divided in half by the ring road (a high-frequency road connecting Messina Center with the motorway), comprises 280 low-rise houses built during the Fascist era. From 2024, this area will be involved in a pilot project of true urban regeneration (Ca-Bis) that will involve the inhabitants in an attempt to create small ecological neighborhoods equipped with services and spaces for enjoyment and socializing. There are no specific data for the populations of Camaro and Bisconte as individual entities, as they are neighborhoods of Messina. The IC La Pira/Gentiluomo school, which welcomes students from the Camaro-Bisconte neighborhood, is divided into four complexes and attended by 691 children aged between 3 and 14 (nursery, primary and lower secondary school), divided into 45 classes with an average of 15 children per class. The socio-cultural environment in which the school operates reflects the problems of areas at risk: high unemployment, illegal employment, alcoholism, petty crime (drug dealing, theft, etc.), family breakdown, illiteracy, lack of adequate facilities, and widespread mistrust of institutions. There are no family counseling centers, adequately equipped squares and green spaces, cultural, sports or recreational centers in the area. The work activities of the neighborhood's inhabitants are clearly linked more to the world of manual labor and craftsmanship, with characteristics of occasionality and, only rarely, to the intellectual world: in fact, there are very few civil servants and professionals. Most parents live in a situation of social and economic hardship, which does not allow them to offer their children a vision for the future, i.e., that fundamental variable for “growing up” in harmony with oneself. The lack of stimulation, cultural resources and social initiatives pushes young people to consider the street as a recurring meeting place where deviant models and negative values such as violence, oppression and illegality predominate;
- Gravitelli is a district located in the upper part of the (covered) Portalegni stream, about 2 km from the city center. Until the earthquake of 1908, Gravitelli was an agricultural area. After the earthquake, the land along the banks of the Portalegni stream was expropriated and used for temporary barracks for displaced persons, also occupying part of the Botanical Gardens (it represents a small arboretum inserted into the building fabric of the city). In the years following the Second World War, the shacks were demolished and replaced with social housing. Over time, many other private buildings were added, causing the number of inhabitants to rise sharply in just a few years. There are no specific data for the populations of Gravitelli as individual entity, as they are neighborhoods of Messina. The socio-economic and cultural background of the residents is average, as they come from diverse social backgrounds, and there is an ever-increasing number of foreign families. The IC Paino/Gravitelli school, is divided into five complexes and attended by 410 children aged between 3 and 14 (nursery, primary and lower secondary school), divided into 29 classes with an average of 15 children per class. The area includes educational agencies, private gyms, parish groups, sports clubs, and social, cultural, sporting, and recreational facilities and services.

### Process

To analyze the structural dimension of the network, interviews were conducted with the participants mentioned above, through the administration of a structured questionnaire of open-ended questions, inspired by social network analysis (Galligani and Riccardo, 2022). This research perspective has been widely used in the field of social research in recent years (Raab and Kenis, 2009). Indeed, the territorialisation of local welfare, and the increasing participation of public and private sector partnerships, have increased the collaborations, both in terms of intervention and project activities, of the organizations that are part of the network. The purpose of the interviews was to stimulate the interviewees to report concrete key episodes that involved them, regarding local welfare and the territorial network. A total of 27 interviews were conducted among the various subjects/representatives, chosen one per organization. Participants were asked to respond qualitatively to a series of open-ended questions, while in other cases they had to summarize their answers using key words and/or images. The perspectives analyzed were twofold: to assess the impact of the Mapping process on the network, in terms of building local welfare; to highlight the role of the network in terms of infrastructure. For these reasons, a specific section of the questionnaire required the respondent to dwell on: (a) the links with other local welfare actors and the quality of these links, both in terms of intensity and the tools used to implement these contacts; (b) the relationships between the actors in the community, both before the network was set up and at subsequent stages. This aspect is important because it made it possible to understand what impact the Mapping experience had in widening and strengthening pre-existing relations between the various actors. And at the same time it is useful to understand how these collaborations can foster local welfare infrastructural outcomes, even after the conclusion of the Mapping project. The questionnaire questions investigating this are the following:

- Question 3: Which community actors did you know and collaborate with before the Mapping project?
- Question 4: Which actors did you get to know by participating in the Mapping project?

This analysis was carried out approximately halfway through the Mapping project, and the results were compared with other qualitative studies (e.g., documentation available on new projects, agreements and educational pacts between the various actors) conducted during the post-project evaluation phase, 6 months after the project's completion, at a feedback meeting attended by all the actors involved. In a final survey form, during this occasion, the following questions was asked:

- Question 5: Which actors did you continue to collaborate with after participating in the Mapping project?
- Question 5 (a): Describe, for each actor, what type of collaboration was created after the Mapping project? (co-design, educational agreement; convention; agreement, etc.)

Therefore, although it cannot be considered a true longitudinal study, it was possible to reconstruct a longitudinal evolution of the network. Compared to the initial situation, the Mapping project highlighted the “sustainability” of the network, in that, cooperative skills were built that remained after the end of the project and served as infrastructure for future projects, relying on tools and practices tested during the Mapping project.

### Meaning

Through these questions, it was possible to gather information qualifying each knowledge and collaboration between the various actors, and aim to reconstruct the pre-existing links between the network's nodes and how the network's constitution itself has impacted the individual nodes' ability to build and exploit collaborative relationships. In fact, the analysis of the data collected was based on network analysis, both of participatory processes and the construction of local welfare, and of knowledge within the network. It was possible to highlight these patterns of interaction that connect the different actors in the network, influence their behavior and modify their network structure. Was used UCINET, an effective software for providing metrics that can be used to characterize nodes and entire networks (Borgatti et al., 2002). The choice of the UCINET programme is justified by the substantial number of procedures (statistical and non-statistical) available in it; this set of procedures makes it the most comprehensive and up-to-date analysis tool compared to others, which are characterized by greater specificity, to the extent that it is probably the best known and most widely used programme for the processing and analysis of relational data. The software offers a large number of metrics that can be used to characterize entire networks and node positions within networks.

The use of this software can be linked to Grounded Theory (Glaser and Strauss, 1967), facilitating the development of a theoretical model firmly grounded in the text, and capable of producing scientific knowledge by making the categories of analysis interact with the meanings constructed by the subjects interviewed (Muhr, 1997). Through the continuous and repeated reading of the interviews, linked to a coding grid corresponding to the objectives of this research, a cyclical and repeated process of analysis, comparison and interpretation was implemented, enabling a systematic approach to the textual data. Network analysis allowed us to examine the relationships that a single actor, or several actors, have with other individuals, groups or organizations in an environment (Wasserman and Faust, 1994). We were thus able to describe the structure of local welfare (Wasserman and Faust, 1994), highlighting the strength, direction and complexity (or number) of embedded ties.

In order to perform social network analysis, the first step was to archive and organize the data so that it could be prepared for network analysis. To this end, a square matrix (*n* × *n*) was created, where each cell (*i* and *j*) represents the relationship between node (*i*) and node (j). The data was entered into the matrix directly, by typing the node labels into the colored cells of the first row and first column, while the relationship information was entered into the other cells. To ensure data reliability, the researcher carried out text transformation operations: elimination of empty forms (articles, prepositions, conjunctions, etc.); attribution of equivalences (through the control of synonyms and pronouns); control of meaning polarities (positive, negative and neutral); polarization; and threshold cutting. With regard to the information in the report, weighted values (from 0 to 5) were assigned to indicate the presence or absence of a relationship, and the strength and frequency of that relationship, in descending order. Specifically, the questions in the questionnaire that investigated these aspects were as follows:

Question 3: Which community actors did you know and collaborate with before the Mapping project?

Question 3 (a): For each actor you mentioned above, how would you describe this collaboration using a scale from 1 to 5 (where 0 stands for no collaboration and 5 for constant collaboration)?

Question 3 (b): Could you briefly give some examples of collaboration (indicating: context; issue/problem; duration; actors involved; interventions/objectives; people reached)?

By visualizing the matrix, we can see that it is a weighted matrix whose numerical values indicate the “closeness” of the relationship. Since a large number of algorithms were available to measure the properties of actors and networks, binary data were used to facilitate the analysis procedures, so that the data expressed as numerical values were converted by the researcher into binary data using the so-called cut-off value procedure (>, 3 ≥, 3 = 1 and o ≤ , o < 2= 0). Before moving on to the analysis procedures, a binary matrix was therefore constructed to recode the data (dichotomization of data), transforming the numerical values into binary values. Once the data had been organized, it was possible to follow different network analysis strategies based on the specific objectives set by the researcher. It was decided to analyze certain descriptive network measures following two main analysis strategies: (1) identifying the degree of network cohesion (group cohesion) and network subgroups (subgroup cohesion); (2) identifying the position of subjects in the network.

Once the matrix had been created, it was possible to graphically visualize the social network.

With respect to the properties of the networks we used the main measures: density, which indicates the degree of cohesion of the network and is calculated by comparing the number of ties detected with potential ties in a network; centralization, which expresses the degree of unevenness in the distribution of the centrality indices of the actors (with the degree centrality and betweeness centrality indices); reciprocity, which measures the tendency of actors to reciprocate ties in a bi-directional manner; connectivity, which describes the number of nodes and ties. Two centrality indices were considered significant: degree centrality and betweenness centrality. The first measures the number of links involving the individual node inbound and outbound (with a normalized value as a percentage ratio), whereby an actor will be more central the more links involve them. The degree level (in particular the incoming one) is an indicator of the social capital of the individual actor and their ability to be a point of reference (considering their high level of knowledge) for other members. The second, on the other hand, represents the frequency with which a node is found on the shortest path (geodesic distance) that connects each of the potential pairs between the nodes that form the network (passing through other nodes). This measure represents the ability of a node to perform a brokerage function between the flows of resources and information passing through the network by virtue of its interposition of the different paths connecting the other actors.

### Limits

It has to be said that we will not be able to specifically discuss the part concerning the laboratory/didactic Mapping experience here, so as not to extend the article further. This part has in fact been dealt with separately in another article (on this subject see Carbone, 2025).

We are also aware of the limitations of this research, which lie in the fact that it is not possible to implement a generalization following a single case study. But this does not detract from the fact that this case study can take on a broader cognitive value with respect to the specific study context (Stake, 2008). By creatively re-interpreting those results in other situations and contexts, they can be re-applied in other contexts of high similarity. In our opinion, this research assumes a theoretical relevance, as it investigates the issue of local welfare that has been increasingly explored in recent years; a pragmatic relevance, as it provides useful insights that could be used as a basis for future empirical research and new research experiences.

## Results

In re-elaborating the results, we decided, for analytical purposes, to break down the treatment of the Mapping project into two sub-cases representing the two Districts in which the project operated. We read the structural constituent elements (governance, social actors, local authorities, agreements, etc.), and the procedural ones (collaboration, co-design, sharing, etc.) found in the two sub-cases (Case 1 and Case 2), and analyzed how the development methods and network dynamics support local welfare.

### Case 1° District IV

The partnership of Case 1° was composed of 13 partners (IC Piano/Gravitelli, University of Messina, USSM, ASP, Diocesan Caritas, Church of Gravitelli, Cooperativa Comunità Minori; Comunità Cristo Re, Collereale Casa Riposo, Palestra Gym, Messina Social City, Lions Club, Libreria Bonazinga).^1^

Analyzing the network structure, it is noted that there are two elements that act as load-bearing arches of the scaffolding of this network, namely the University of Messina and the IC Paino/Gravitelli. This structure depends on the result of the project management role carried out by the IC Paino/Gravitelli in support of the University. The other actors in the network, in particular the Associations, assume a peripheral position, not collaborating directly with each other and remaining connected to the network through links with the “load-bearing” actors. From this mapping, it emerges that the network, although substantially interconnected, does not present network gaps, but is still quite weak. In Table 2 it is possible to see the properties of the network: parameters, centrality, and network impact. The density is equal to 13%, therefore it is very low, and this depends on the effect of the overall lack of ties that affect each node (on average 2.8 if we compare the average degree). Nonetheless, the network is substantially balanced (centralization at 55%) and moderately solid (see reciprocity at 63% and connectivity at 70%).

**Table 2:** Network parameters, subject centrality and network impact Case 1.

**Network properties**					
	**Medium grade**	**Density**	**Centralization**	**Reciprocity**	**Connectivity**
Values	2.8	13%	53%	63%	70%
**Centrality indices of the 5 predominant actors**					
**Partner**		**Degree centrality**		**Betweenness centrality**	
University of Messina		25		170	
IC Paino/Gravitelli		20		72	
Common Messina		19		101	
IC La Pira/Gentiluomo		16		92	
Lions club		14		32	
**Difference between pre and post mapping dyad bonds**					
**Ex-ante mapping dyads**		**In Itinere mapping dyads**		**Post mapping dyads**	
25		33(+34%)		29	

Source: Analysis of collected data and own processing.

The table shows the presence of a circle of actors that, together with the University of Messina and the CI Paino/Gravitelli, demonstrate sufficient relational capacity to support the network. In addition to the two leading actors, considering degree centrality, a good performance of the La Pira/Gentiluomo CI emerges, as well as a fair value of betweenness for the network's municipal administration. This points to a good relational potential of various subjects who, through the Mapping project, had a further opportunity to forge new ties with respect to their pre-existing ones. The mobilization of the municipal administration throughout the mapping project has not only enhanced the network, but also the territory in general, and consolidated certain roles.

“*The difficulty for us has always been to communicate with the municipal administration because some neighborhoods and the people who live there are often difficult to reach. So today, getting closer to those areas, thanks in part to agreements with the municipality, is a positive response that encourages further participation and produces positive effects.”* (Minor Communities Interview)

The Mapping project increased the number of dyads by 34% compared to the ex-ante Mapping collaboration network.

**Figure 2:**
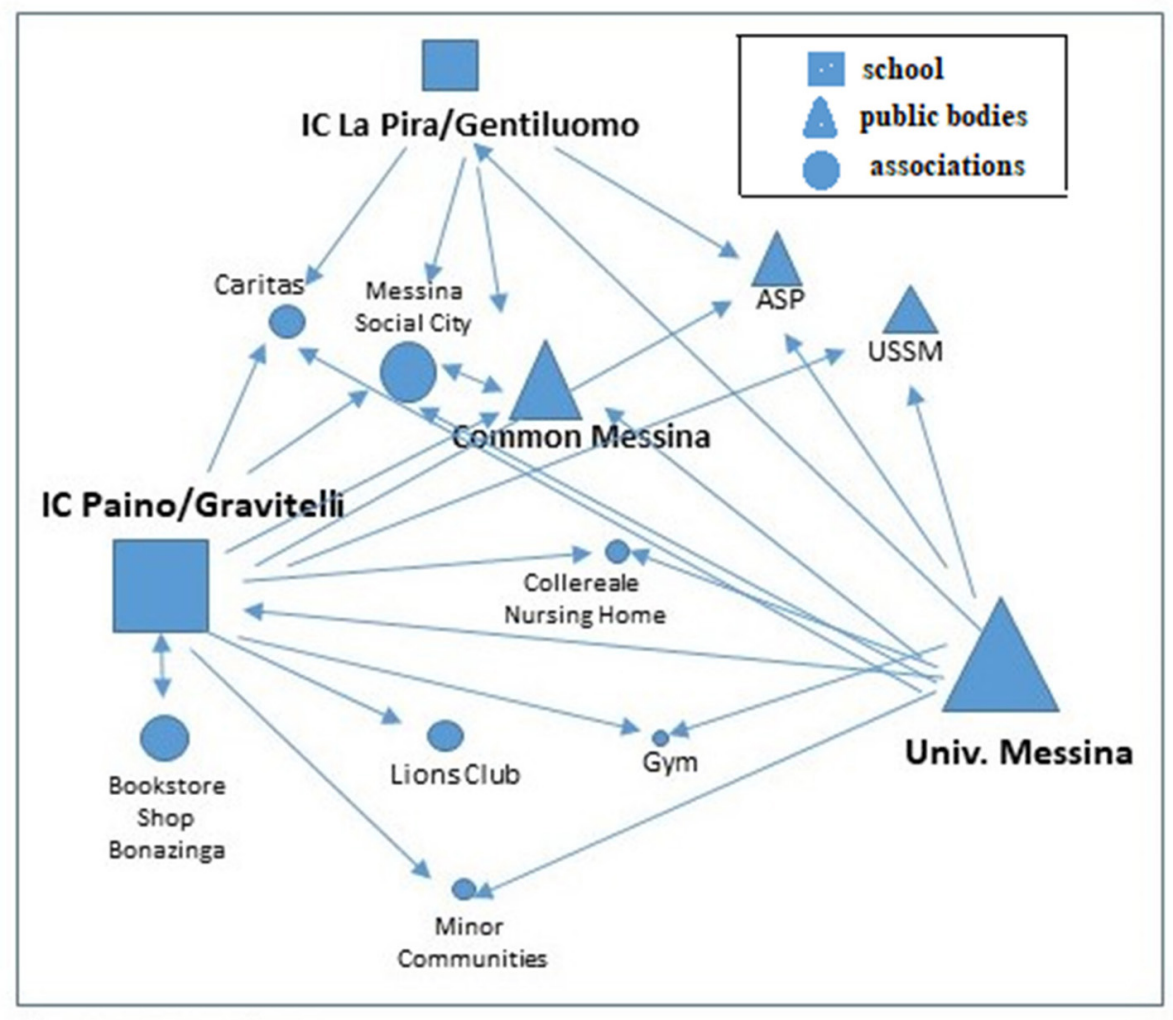
The Case's 1° partnership network. Source: own processing.

**Figure 3:**
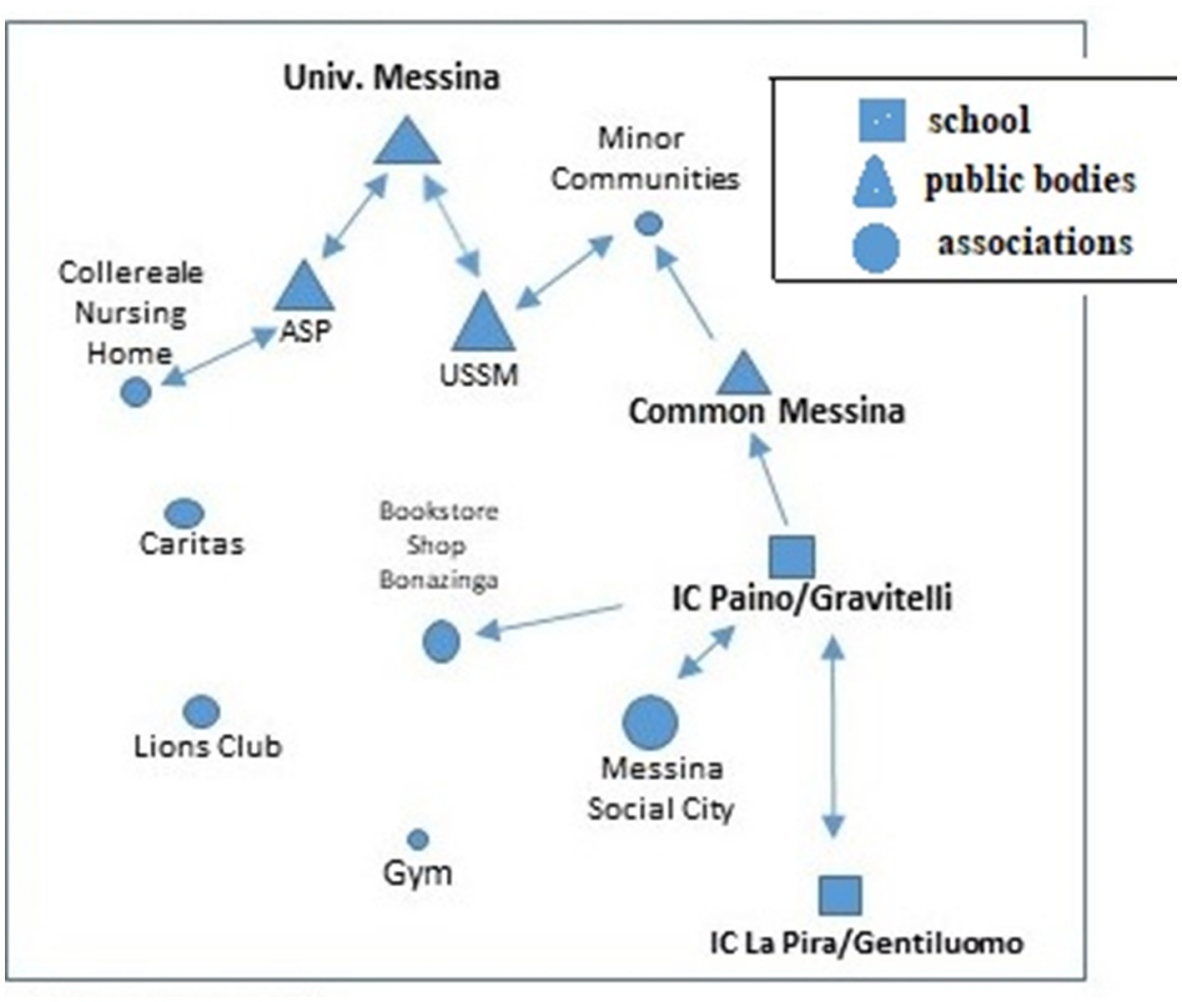
The post-project partnership network 1 ° Case mapping. Source: own processing.

The Paino/Gravitelli school benefited greatly from this increase. In essence, the local welfare of Case 1 is driven by both the University of Messina and the Paino/Gravitelli CI. In this case, the two CIs also interact with each other. With the Associations, on the other hand, direct collaboration channels prevail, making the Paino/Gravitelli CI essentially a beneficiary of the network's activities, rather than a protagonist. Looking at the number of collaborations, established since the Mapping project, or prior to it, only 25 dyads out of a total of 33 can be considered “durable.” If we compare Figure 2, where we have depicted the project network with the potential post-project network in Figure 3, we can see that the partnership is now in danger of becoming fragmented and impoverished.

In conclusion, we can effectively state that the Mapping project proved to be highly effective in pursuing the process dimensions for the launch of the local welfare infrastructure. Although it cannot be considered a longitudinal study, as far as it was possible to ascertain with respect to the initial situation, the Mapping project highlighted the “sustainability” of the network, in that cooperative skills were developed that remained after the end of the project and served as infrastructure for future projects, relying on proven tools and practices. Within the school context it managed to involve families and teachers, succeeding in disseminating democratic principles and grasping the value of the social capital existing in the various actors in the area.

This recognition by schools of their role as entities ready to take on the responsibility of being the hub of the local welfare network is clearly expressed:

“*Together with the school and around the school, it is possible to create continuous interaction with other opportunities for associated experiences outside the institution itself. This strategy must involve private and public bodies, the Third sector, the market and the population.” (School Management Interview Case 1)*

In this sense, it is the school that is conceived as an open school (Rotondi, 2019), ready to work by enhancing synergies with the intermediate actors around it.

Also other actors explain the importance of sharing a common language and values among all members:

“*The network must also build alliances with families and school sharing common elements and values. Therefore, this alliance must be built together, at the local level. This is why we participated in the mapping project and why we were also present before, with the schools, and will continue to be present afterwards.” (interview with the USSM social worker)*.

Along this path, the partnership activated a reflexive process on the actions that, in addition to having led to the construction of the network, may be instrumental in continuing the initiative. The graph Figure 3 shows the willingness to continue using a one-to-one collaboration model in the future, which does not actually reinvigorate the network. In this sense, local welfare still appears to be projected toward informal forms of collaboration, where mutual knowledge remains, and more methods of collaboration are potentially to be shared, but without a solid infrastructure behind it to guarantee continuity.

### Case 2 District III

The partnership of the second case was composed of 12 partners (University of Messina, USSM, IC La Pira/Gentiluomo, ACR Camaro Calcio, ASP, Caritas Diocesana, Chiesa San Paolino, Cooperativa Comunità Minori; Municipality, Associazione Ciclope; Camaro Urban Park, Save the Children, Messina Social City).

**Figure 4:**
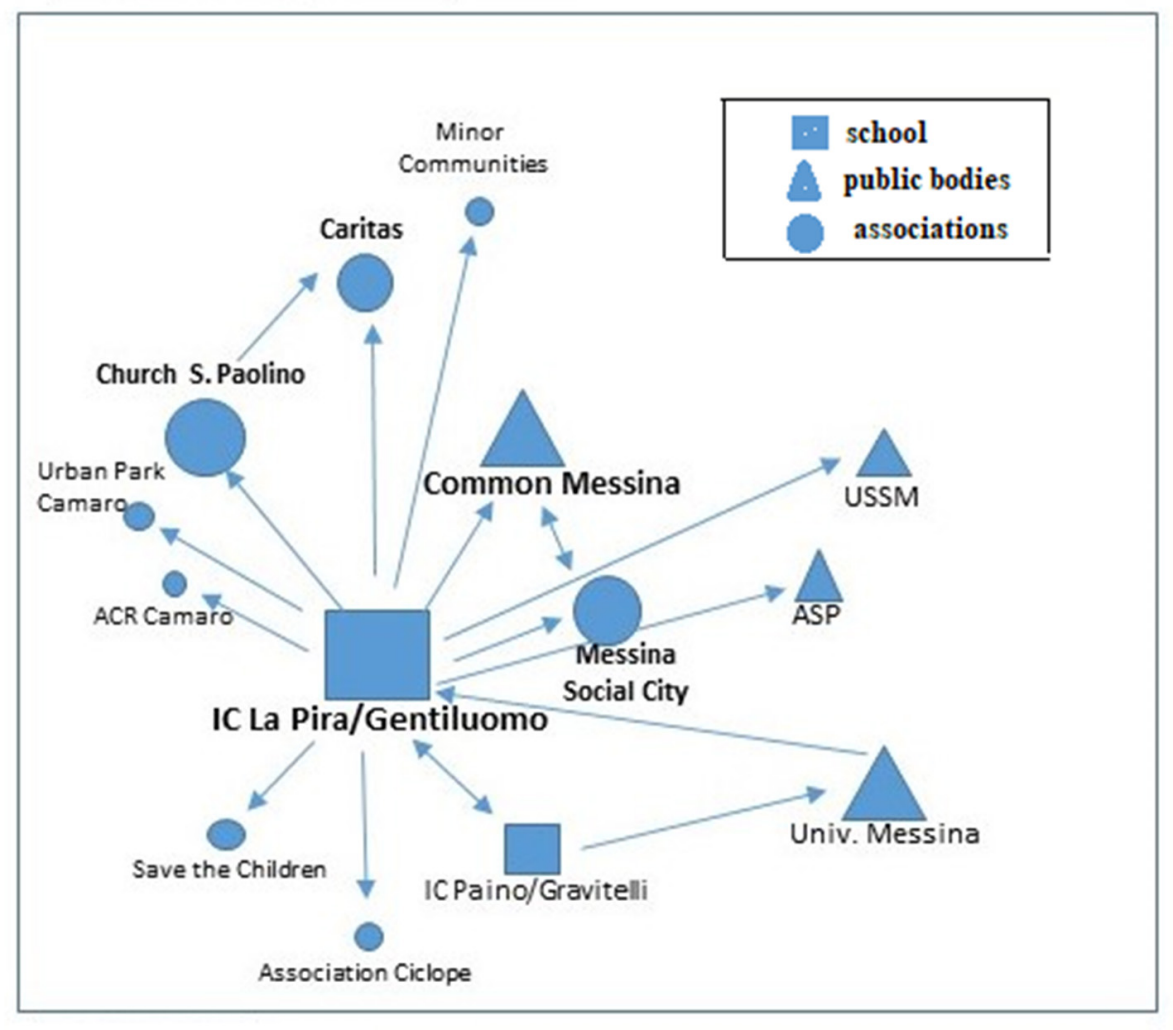
The Case's 2 ° partnership network. Source: own processing.

Looking at the conformation of the overall network (Figure 4), it emerges that although Associations and Cooperatives make up the majority of partners, the party with the most links is the school. In fact, the Associations and Cooperatives have a lower network weight and interact with each other. The network thus appears less solid and rich, but sufficiently dense and well-connected, to a much greater extent than Case 1. Looking at the properties of the network it is possible to find an average configuration, to be considered neither too weak nor too excellent (Table 3).

**Table 3:** Network parameters, subject centrality and network impact Case 2.

**Network properties**					
	**Medium grade**	**Density**	**Centralization**	**Reciprocity**	**Connectivity**
Values	3.2	26%	57%	64%	83%
**Centrality indices of the 5 predominant actors**					
**Partner**		**Degree centrality**		**Betweenness centrality**	
IC La Pira/Gentiluomo		19		50	
Messina social city		18		76	
Church San Paolino		16		28	
Caritas		13		25	
Common Messina		13		14	
**Difference between pre and post Mapping dyad bonds**					
**Ex-ante mapping dyads**		**In itinere mapping dyads**		**Post mapping dyads**	
28		37(+44%)		35	

Source: Analysis of collected data and own processing.

The density is 26%, while the average grade is 3.2, to be considered neither weak nor excellent. Good reciprocity rates (64%), and especially connectivity at 83% are evident. This shows a substantial solidity and balance of the network. This can be seen from the good distribution of the actors' centrality indices. The Messina Social City demonstrates a greater capacity for intermediation in terms of betweenness, and its strength is shown above all in acting as a connector between the school and other bodies and associations. From a networking point of view, on the whole, the evaluation appears substantially positive. This fair networking potential is also evident from the percentage increase of dyads thanks to the Mapping project, by 44% compared to the ex-ante situation. With respect to networking, the Mapping project was able to involve other associations and cooperatives not initially foreseen. The IC La Pira/Gentiluomo school was more inclined to promote post-project networking tools.

In conclusion, the Case 2 network shows the capacity to become a “system,” activating a process of progressive growth and relaunching of collaboration with other organizations and Associations/Cooperatives. The consolidation of this local welfare project is demonstrated in the various activities following the Mapping project that involved the network, giving all the actors involved the opportunity to get to know each other and consolidate a common language and shared vision. One strategy could be to adopt tools that enable infrastructure to be built and prevent the aggregation work begun with the Mapping project from being lost. This has led to some actors wanting to launch other micro-projects to relaunch the local network:

“*We started a ‘summer school' with the school. We asked ourselves: what kind of activities would we like to do? Who do we want to target? Families, schools and children were our target audience and also our strongest allies.” (Camaro Urban Park)*

### Comparison between cases

What emerged, upon initial analysis, is that the mapping programme (which we can consider the macro-case) determined two different configurations (strategies-context) in the two contexts, as if they were autonomous entities, almost belonging to separate projects. It was therefore necessary to undertake an overall assessment of the programme, operating a form of comparison and theoretical synthesis, establishing a dialectic between the sub-project and the macro-project, which is substantiated during an analytical process, implementing a triangulation of evidence and explanatory hypotheses in the construction of the case (Carter and Sealey, 2009). Tracing the technique of systematic comparison in the Grounded Theory Method (Flick, 2019), the triangulation took place through a constant and interactive process of construction and refinement of interpretations, through the search for performing and constituent elements of local welfare, determined by specific observational parameters adopted, constituting specific views of the phenomenon (Fielding and Fielding, 2008). Let us begin with an initial comparison of the structural-network configurations of the two different partnerships in order to delineate the different governance arrangements and highlight the possible contribution of these configurations in determining infrastructure outcomes. Comparing the different measures found, it can be seen that these two cases both tend not to be over-centralized, in fact the values are not too far from 50%, and far too dense, in fact the highest value is 26%. This explains how, in Case 2, there is greater connectivity, which is to be understood as the network's ability to resist the risks of fragmentation, determined by the large number of links that favor the solidity of the networks. Ultimately, two different patterns emerge from the network measurements: on the one hand, Case 2 shows denser and more connected networks; on the other hand, Case 1 with more centralized and weaker networks, but with relatively high levels of reciprocity, characterized by looser conformations but relatively stronger ties.

Concerning the trend of the number of dyads among the actors according to the different project phases (ex-ante/in itinere/post), Case 2 is characterized by a greater richness and solidity of the partnership networks and a higher attitude to involve external actors. In addition, Case 2 shows a greater impact of the project implementation in enlarging the network (the number of dyads) compared to the ex-ante situation. All in all, Case 2 demonstrates a significantly higher attitude toward networking.

**Figure 5:**
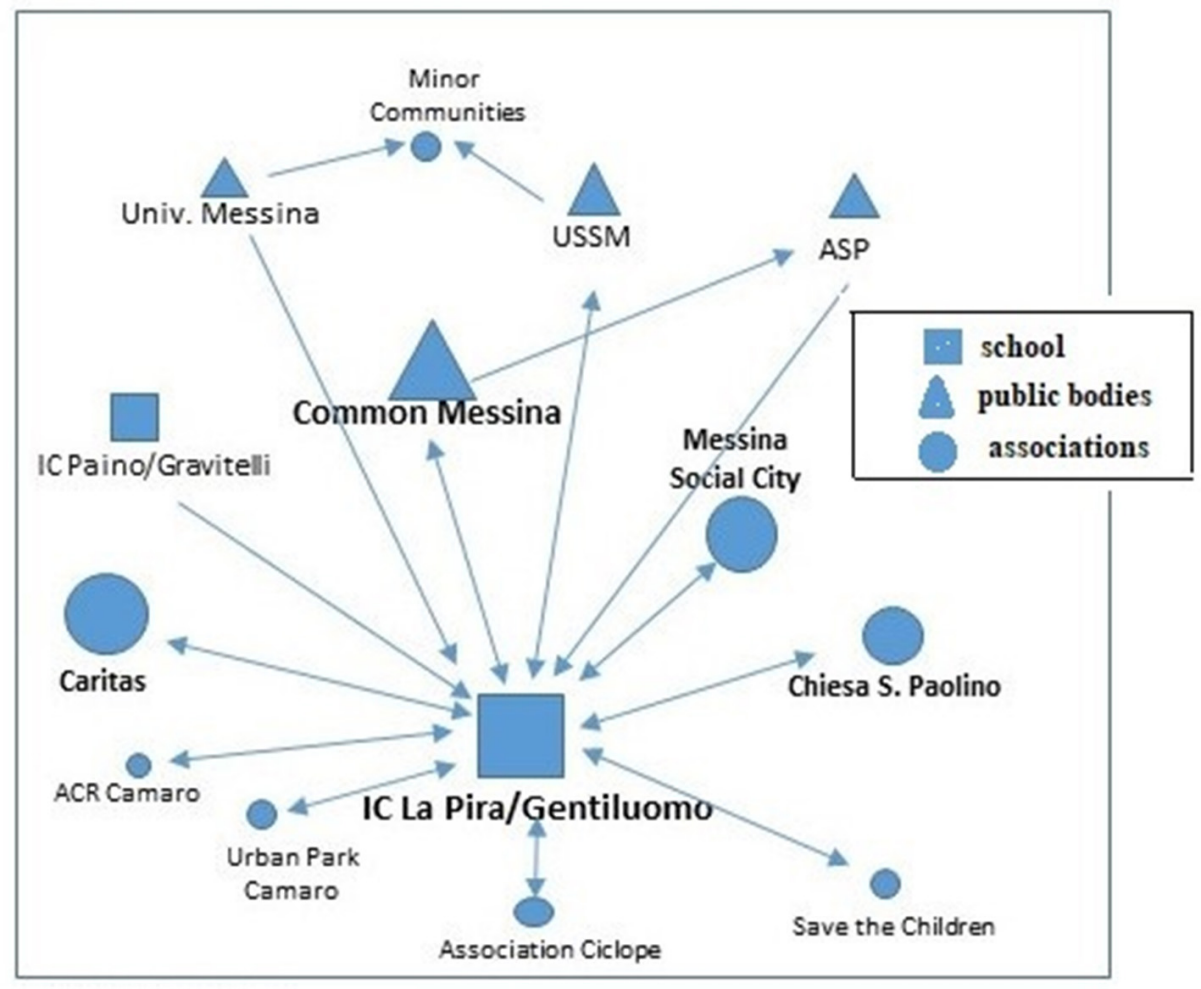
The post-project partnership network Case 2 ° Case mapping. Source: own processing.

This aptitude is then further confirmed by the positive difference in the number of dyads between pre-project and post-project Mapping, which sees the number increase by +7%, thus demonstrating a high impact of the project in expanding local welfare. In contrast to this, Case 1 has a value that has certainly not demonstrated a high aptitude for networking, beyond the already established collaborations. Networking solidity is not in itself sufficient to guarantee the sustainability of the collaborations that give rise to the partnership networks. What can be deduced from this initial analysis is that only by looking at the trend and quantity of the dyads, rather than the richness of the network, can the actual sustainability of the network be assessed. And where the municipality and the school take a central role, it is able to facilitate the infrastructure of the network in the post-project period. Let us now place these reflections on the characteristics of the network alongside those on the processes and the role played by the various actors, analyzing the two cases longitudinally in order to identify the most salient themes for each. The network of Case 1 appears in the first instance as a network with governance restricted to a single actor (or a few actors) in which decision-making power is concentrated in the hands of a few actors. In this way, decision-making power is more concentrated on the individual actors, and it is easier to achieve the set goals and objectives, limiting misunderstandings and conflicts. The Case 2 network is different, where there is a more participative governance, based on a diffuse interactional and decision-making process, in which no one actor prevails over the other, and decision-making power is distributed among a multiplicity of actors. This makes the network more solid because it enables it to survive the eventual failure of a central actor. Moreover, in this model there is a greater inclusiveness and a high degree of internal legitimacy, where a high level of trust and consensus between actors sharing the same objectives is necessary for it to function. In both cases, the role of the University of Messina should be emphasized, and specifically that of the researcher who conceived the Mapping project, pursuing an initiative capable of experimenting with the circularity between education, research and the Third Mission. The latter can be considered a favorable condition for forging links between the University and the local area. In this context, the role of universities in the production of knowledge and in collaboration with organizations and various social actors operating in the territory takes on particular importance, activating interesting mechanisms for the exchange of knowledge and practices, as well as the unfolding of a concrete collective social process characterized by a significant level of complexity in terms of social infrastructure. In particular, we can consider public engagement as an activity oriented toward a circular and participatory mode of interaction between different subjects (Warnke and Brüggenwirth, 2022) who are united by one or more shared goals. In the Mapping project, the SCIPOG department, through a written agreement, played a coordinating, facilitating, continuity and liaison role with the territory, convening meetings and systematizing the various proposals coming from the members of the network. This network is sustaining itself because the individual nodes have found it beneficial to converge on activities without any initial external constraints (such as a call for funding), but rather focused on the idea of co-constructing a space for discussion on experiences and practices (Figure 5). Based on these reflections, it is interesting to note how the University can act as a catalyst and driver of new relationships between local authorities, without however constituting an essential and indispensable element in the subsequent dynamics of the network. Furthermore, the local authority's protagonism emerged as a positive element in both cases analyzed. In fact, the administration's awareness of the importance of intervening within the local welfare system, thanks to the schools' proactiveness, with consequent participation in supporting the municipal authority in a leading role, is based above all on past experience of collaboration.

*The municipality's participation becomes a resource for the network and can make the project more “solid,” including through funding and new human resources to invest*.

“*As a conscientious administration, we are physically present to listen directly to people's needs. We do not shy away from our commitment and we deploy all the resources we have, both human and economic.” (District President Interview)*

The quality of the collaborations during the project fostered further consolidation of the network. The impact of the involvement in co-design work with the municipal authority also became a means of strengthening the collaborative bond. International research, confirming this, has also found that this new paradigm of collaborative welfare places municipalities at the center of strategic direction, requiring coordination skills, management innovation and openness to cooperation with citizens and local communities, mirroring experiences of participatory governance also observed in Great Britain and Canada (Rhodes, 2012; Bovaird and Löffler, 2012). The continuity and intensity of the co-design activities of the individual initiatives made it possible, not only to realize better activities, but also to strengthen or establish relationships between the actors, building a project community determined to build lasting collaboration. Essentially, thanks to the opportunities that emerged subsequently, the partnership was able to increase its capacity to involve the local area itself, increasing opportunities for co-designing the network:

“*The project allowed us all to achieve more difficult targets. Through this approach, we entered into the lives of families, working with children and parents themselves, together as a network, not individually. At the network level, we have laid the foundations to continue with work that connects people in the local area and services.” (Save the Children interview)*.

Finally, with regard to partnership permeability and external networking practices, in both cases valuable strategies were initiated to make the action more effective with respect to the beneficiaries, by differentiating the contexts of action and the professional resources available. This traction of the private social sector, which is essential for the network's vitality and sustainability, requires a decisive strengthening of ties with the municipal authority and the school.

This process of traction of the private social sector is the most effective configuration in terms of territorial involvement, since it enhances the Third Sector's capacity for local animation and thus also the permeability, inclusiveness and exponential growth of the network, as well as in the provision of social services (Salamon and Anheier, 2013), contributing to resilience and social cohesion, as observed by Evers and Laville (2004) in Europe. In Case 2 the outcome of this process of traction gave rise to a consequent relaunch of the project, whereas in Case 1 the conformation of the network experimented in the Mapping project remains static, awaiting future developments. In order to explain the differences that determine the varying evolution of networks, after analyzing the post-project structural dimension, attention was focused on the role played by the different types of actors in the network. What emerges, rather than the richness of the network, is that the sustainability of the network in the post-project phase depends on the number of dyads (out of the total) that have the school at the top. In fact, in Case 2, there was more evidence of network infrastructure in the post-project phase. A relevant demonstration can be found after analyzing, at the end of the Mapping project, the documentation (e.g., projects, agreements and educational pacts) from which some relaunches implemented by the school in Case 2 can be inferred. New local educational micro-project practices were identified, with the school playing a central role in co-designing not only with some social actors who had experimented with building social infrastructure through the mapping project, but also involving new local actors. This evidence suggests a more general reflection on the role played by the different categories of actors and how their behavior can affect the social infrastructure process. Reduced activism on the part of the school, relegated to a secondary role, can become, as in Case 1, a factor of weakness in the social infrastructure process. On the contrary, activism on the part of schools facilitates continuity of funding, availability of facilities, social legitimacy and direct contact with beneficiaries (families/population), as well as greater mobilization toward external actors.

## Discussion

We can conclude by stating that the Mapping project was able to actively involve, in both cases, organizations and Associations/Cooperatives, consolidating and sharing a common vision on community planning. a local welfare system doesn't exist in isolation or as something simply imposed from above. Instead, it finds its legitimacy and raison d'être (reason for being) in the context of a territorial development project. This project is co-created with the people who choose to actively participate in it. These are not passive recipients, but individuals who voluntarily assume responsibility for implementing a participatory and collective project of transformation. In this framework, the community isn't just a beneficiary of social services; rather, it plays an active role in shaping the direction of the welfare system. The process is described as transformative, meaning it is about creating meaningful changes within the community. This transformation is not just material but also symbolic, as it involves the shared construction of meaning and values that define the actions within the territory. Through significant investment, the Mapping project created a certain momentum on the network, investing heavily in work and co-design. The partnership was also able to create specific tools and projects capable of strengthening and relaunching the social network and local action in order to consolidate this high level of participation.

In this sense, the Mapping experience demonstrates the theory that where a strategy of involvement and infrastructure of a network is created, it is possible to implement it in a coherent manner (actors involved, practices, interventions) creating the prerequisites of structure, process and sense of community. This leads the local network to infrastructure itself with ad hoc tools based on the experience gained. Subsequently, this infrastructure will serve as the basis for the construction of a renewed local welfare, capable of taking care of its own local development project. The two case studies analyzed show that in order to build an effective local welfare, it is not enough just to put together a local community network around a shared identity vision, it is necessary to make the different actors learn to know each other, dialogue and work together, going beyond the territorial weave, and aiming at the rooting of the infrastructure. It is precisely through knowledge and collaboration that all actors can understand the importance of their own contribution and participation, which is founded in a unique collective value, as Meo (2022) would say, where the capital that can be made available to the territory is rediscovered.

In this project, the University of Messina proposed itself as the driving force behind relations between local authorities, without constituting an indispensable and indispensable element in the network's subsequent dynamics. This dynamic found in this research finds correspondence in various international contexts, where the literature has analyzed the growing role of local authority and nonprofit organizations in managing social services and building resilient collaborative networks. Osborne (2010), Emerson et al. (2012) had highlighted that this coordination and cooperation between public, private and citizen actors increases the effectiveness of services and the capacity of communities to address complex problems. The data collected and processed show that experiences such as this Mapping exercise are not enough to consolidate and structure the network, but that continuous support is needed from certain actors, such as the local authority and schools. In fact, the risk is that, where the activity of some central actors (e.g., the University of Messina) decreases, it will be difficult to keep the collaboration alive and there will be a return, in line with what De Ambrogio and Guidetti (2016) stated, to each working individually with their own small networks, as in Case 1. Therefore, on the one hand, it would be important for the local authority itself to strengthen its protagonism, making use of the link with other institutions, and increasing the moments of meeting and co-planning with other actors. In this perspective, the good response to the Mapping project becomes a stimulus for the municipal authority to actively engage on policy, playing the role of ensuring interventions, providing places, services and infrastructures, and openly manifesting a political will to support the co-design activity (Rossi-Doria, 2020), also by investing a greater financial endowment, in order to become a more central node in the network. Through this commitment, as noted by Bovaird and Löffler (2012), the co-production of services and interventions not only improves efficiency, but increases legitimacy and trust in local institutions.

But it will be strategic, as De Ambrogio and Guidetti (2016) suggest, to involve the schools more closely, enhancing the potential for participation inherent, for example, in interclass meetings, class councils and school boards. This intentional projection of collaboration should be emphasized, which is voluntarily manifested by the interviewees, as a choice and awareness of adhering to the recognition of a road to be traveled united (Zamengo and Valenzano, 2018), taking the first steps toward a process of change of a local welfare system (De Ambrogio and Guidetti, 2016), but which needs, in order to be consolidated over time, concrete political support from the municipal authority (Rossi-Doria, 2020; Luongo et al., 2022). Finally, we can say that this research, in addressing a very important issue that encompasses sociology and local welfare social policies, outlines a theoretical and methodological framework for the analysis and evaluation of local welfare networks as forms of social infrastructure. Today, it is important to ask ourselves: what could be the lines of development and cooperation in local welfare? From this perspective, the process of building local welfare networks, as reported in this research, can also be taken as a specific example of a more general spread of social infrastructure practices, understood as the development of intangible structures (relational networks) useful for increasing the inclusiveness and effectiveness of current welfare systems. The theoretical and cognitive proposals of the research, enhanced in an applied context (specifically in the field of social welfare policies), can represent an original contribution. Therefore, the perspective adopted in this research emphasizes the need to enhance the endogenous resources of a territory in order to promote a model of solidarity built mainly on the immaterial and intangible resources of a territory (social infrastructure), constructing interconnection networks that become the human-relational, organizational and socio-technical pillars of local welfare. This advanced local welfare model, according to Rhodes (2012), is based precisely on complex networks of relationships between institutions and resources of territory, capable of strengthening local accountability and the personalization of services.

These networks are not just a product that is limited to participatory logic, but encourage the enhancement of a process of social aggregation capable of changing the existing situation of immobility and generating local socio-political growth.

## Conclusion

The two cases analyzed confirm that participatory processes, such as Mapping, can not only strengthen welfare networks, but can also acquire a transformative value to initiate a process of social infrastructure development capable of changing the local welfare system. It is not a question of identifying an ideal network structure to build, but rather of analyzing which structures are most effective and sustainable over time and, more generally, the role played by the different categories of actors. In essence, the path to further strengthening social infrastructure processes and consolidated local welfare could involve strengthening the role of schools, including by considering a specific system of cooperation between schools in the area. Ultimately, with regard to general theories on local welfare and social infrastructure, Case 2 demonstrates that, starting from a strong push for networking and co-design, the Mapping project has been able to translate these intentions at the local level, generating a significant impact on the network. To consolidate this high level of participation, the partnership, led by the school's activism, was able to develop specific tools and project ideas capable of consolidating and relaunching the network in the post-project phase. In this sense, the theory has been proven that developing a strategy for the involvement and social infrastructure of a network, implementing it consistently by creating the structural and process conditions that lead to the network's infrastructure, and placing specific categories of actors at the helm, constitute the foundation of a renewed local welfare system. Finally, this reconceptualization study may have important implications in terms of social policies, as it suggests that for local welfare to be effective, it is not enough for policies to be designed and implemented by central institutions; it is crucial that local actors and communities are actively involved. Social policies should therefore encourage the direct participation of all local social actors, supporting the commitment and role of local institutions (e.g., through public funding), but decentralizing resources and services in order to promote organization at the territorial level (through local capacity building and the creation of solidarity networks) so as to better respond to local specificities.

## Data Availability

The original contributions presented in the study are included in the article/supplementary material, further inquiries can be directed to the corresponding author.

## Footnotes

1 In the graph in Figure 2 (and those that follow), the shape of the node represents the type of organization: the square for schools; the triangle for public bodies; and the circle for Associations and Cooperatives. The size of the node, on the other hand, depends on the number of links to which that node is an incoming and outgoing vertex (degree centrality).

## References

[B1] Accorinti M. (2008). Terzo settore e welfare locale. Roma: Carocci.

[B2] Albæk K. Kjaer U. Pedersen L. H. (2019). Local Governance and Social Policy: Comparative Perspectives on Partnerships in Europe. London: Routledge.

[B3] Bartlett L. Vavrus F. (2017). Rethinking Case Study Research: A Comparative Approach. London: Routledge. doi: 10.4324/9781315674889

[B4] Borgatti S. P. Everett M. G. Freeman L. C. (2002). Ucinet for Windows: Software for Social Network Analysis. Harvard, MA: Analytic Technologies.

[B5] Bovaird T. Löffler E. (2012). Public Management and Governance, 2nd Edn. London: Routledge.

[B6] Carbone S. (2025). Participatory training of social work students: a mapping experience. Social Work Educ. 1–19. doi: 10.1080/02615479.2025.2474506

[B7] Carter B. Sealey A. (2009). “Reflexivity, realism and the process of casing,” in The SAGE Handbook of Case-Based Method, eds. D. Byrne and C. C. Ragin (London: Sage), 69–83. doi: 10.4135/9781446249413.n4

[B8] De Ambrogio U. Guidetti C. (2016). La coprogettazione. La partnership tra pubblico e terzo settore. Carocci Faber: Roma.

[B9] Emerson K. Nabatchi T. Balogh S. (2012). An integrative framework for collaborative governance. J. Public Adminis. Res. Theory 22, 1–29. doi: 10.1093/jopart/mur011

[B10] Everitt A. Hardiker P. Littlewood J. Mullender A. (1992). “Epistemology and theory in social work,” in Applied Research for Better Practice. Practical Social Work (London: Palgrave). doi: 10.1007/978-1-349-22265-0_2

[B11] Evers A. Laville J.-L. (eds.) (2004). The Third Sector in Europe: Between the State and the Market. Cheltenham: Edward Elgar. doi: 10.4337/9781843769774.00006

[B12] Ferrera M. (2007). Trent'anni dopo. Il welfare state europeo tra crisi e trasformazione [Thirty years later. The European welfare state between crisis and transformation]. Stato Mercato 27, 341–376. doi: 10.1425/25907

[B13] Fielding J. Fielding N. G. (2008). “Synergy and synthesis: integrating qualitative and quantitative data,” in The SAGE Handbook of Social Research Methods, eds. P. Alasuutrari, L. Bickman, and J. Brannen (Thousand Oaks, CA: Sage), 555–571. doi: 10.4135/9781446212165.n33

[B14] Flick U. (2019). “From intuition to reflexive construction: research design and triangulation in grounded theory research,” in The SAGE Handbook of Current Developments in Grounded Theory, eds. A. Bryant and K. Charmaz (London: SAGE), 125–144. doi: 10.4135/9781526485656.n8

[B15] Fondazione con il Sud (FCS) (2021). Bilancio di missione, 2020. Roma: FCS.

[B16] Galligani I. Riccardo A. (2022). L'analisi delle reti sociali per la valutazione delle comunità educanti nei progetti di contrasto alla povertà educative. Rassegna Italiana Valutazione 80–81, 185–207. doi: 10.3280/RIV2021-080010

[B17] Gangarova T. von Unger H. (2020). “Community Mapping als Methode,” in Partizipative Forschung, eds. S. Hartung, P. Wihofszky, and M. Wright (Wiesbaden: Springer VS), 143–177. doi: 10.1007/978-3-658-30361-7_5

[B18] Giovannetti E. Gori C. Pacini G. (2014). La pratica del welfare locale. L'evoluzione degli interventi e le sfide per i comuni. Milano: Maggioli.

[B19] Glaser B. Strauss A. (1967). The Discovery of Grounded Theory: Strategies for Qualitative Research. Mill Valley, CA: Sociology Press.

[B20] Kazepov Y. Cefalo R. (2020). La dimensione territoriale delle politiche sociali [The territorial dimension of social policies]. Parolechiave 28, 85–99. doi: 10.7377/100538

[B21] Kettl D. F. (2015). The Transformation of Governance: Public Administration for the Twenty-First Century. Baltimore, MD: Johns Hopkins University Press.

[B22] Kumar S. (2002). Methods for Community Participation: A Complete Guide for Practitioners. London: ITDG Publishing.

[B23] Larkin B. (2013). The politics and poetics of infrastructure. Annu. Rev. Anthropol. 42, 327–343. doi: 10.1146/annurev-anthro-092412-155522

[B24] Latham A. Layton J. (2022). Social infrastructure: why it matters and how urban geographers might study it. Urban Geogr. 43, 659–668. doi: 10.1080/02723638.2021.2003609

[B25] Luongo P. Morniroli A. Rossi-Doria M. (2022). Rammendare. Il lavoro sociale ed educativo come leva per lo sviluppo. Roma: Donzelli.

[B26] Meo V. (2022). Facciamo un patto! I patti educative di comunità e la parteciapzione delle ragazze e dei ragazzi. Italia, Milano: Franco Angeli; UNICEF.

[B27] Moreno L. McEwen N. (2005). “Exploring the territorial politics of welfare,” in The Territorial Politics of Welfare (Oxon; New York, NY: Routledge), 1–40.

[B28] Muhr T. (1997). ATLAS.ti 5: The Knowledge Workbench. Berlin: Scientific Software Development.

[B29] Osborne S. P. (2010). The New Public Governance? Emerging Perspectives on the Theory and Practice of Public Governance. London: Routledge. doi: 10.4324/9780203861684

[B30] Raab J. Kenis P. (2009). Heading toward a society of networks: empirical developments and theoretical challenges. J. Manage. Inq. 18, 198–210. doi: 10.1177/1056492609337493

[B31] Rhodes R. A. W. (2012). “Local governance and governance networks in comparative perspective,” in The SAGE Handbook of Public Administration, eds. G. Peters and J. Pierre (London: SAGE), 418–432.

[B32] Rizzuto G. (2020). La ricerca azione e la comunit? educante: uno sguardo critic e una ipotesi di lavoro a partire da esperienze nel centro storico di Palermo [Action research and the educational community: a critical perspective and a working hypothesis based on experiences in the historical center of Palermo]. Tracce Urbane 8, 242–254. doi: 10.13133/2532-6562_4.8.17041

[B33] Rossi-Doria M. (2020). Polis e politiche educative: per una comunità educante. Educazione sentimentale 1, 143–151. doi: 10.3280/EDS2014-021014

[B34] Rotondi S. (2019). “Fondo per il contrasto della povertà educative minorile,” in Intervento a Welforum 2019 (Roma: Inapp).

[B35] Salamon L. M. Anheier H. (2013). “The third world's third sector in comparative perspective,” in International Perspectives on Voluntary Action (London: Routledge), 60–93.

[B36] Stake R. (1995). Case Study Research. Cham: Springer.

[B37] Stake R. E. (2008). “Qualitative case studies,” in Strategies of Qualitative Inquiry, 3rd Edn, eds. N. K. Denzin and Y. S. Lincoln (New York, NY: Sage Publications), 119–149.

[B38] Star S. L. (1999). The ethnography of infrastructure. Am. Behav. Scientist 43, 377–391. doi: 10.1177/00027649921955326

[B39] Triani P. (2014). Quando scuola, territorio e servizi collaborano: l'approccio cooperative nelle organizzazioni. Cittadini crescita 3, 22–26.

[B40] Valenzano N. (2019). Enhancement or empathy education? From pedagogical anthropology to educational practices. Formazione Insegnamento 17(1 Suppl.), 355–362. Available online at: https://ojs.pensamultimedia.it/index.php/siref/article/view/3337

[B41] Warnke M. Br?ggenwirth S. (2022). Waveform adaptation for target classification using HRRP in a cognitive framework. IEEE Trans. Aerospace Electron. Syst. 59, 3695–3712. doi: 10.1109/TAES.2022.3230659

[B42] Wasserman S. Faust K. (1994). Social Network Analysis: Methods and Applications. Cambridge: Cambridge University Press. doi: 10.1017/CBO9780511815478

[B43] Zamengo F. Valenzano N. (2018). Pratiche di comunità educanti. Pensiero riflessivo e spazi condivisi di educazione tra adulti. Ricerche Pedagogiche 208, 345–374.

[B44] Zhu E. Baylen D. M. (2005). From learning community to community learning: pedagogy, technology and interactivity. Educ. Media Int. 42, 251–268. doi: 10.1080/09523980500161395

